# NEOadjuvant therapy monitoring with PET and CT in Esophageal Cancer (NEOPEC-trial)

**DOI:** 10.1186/1756-6649-8-3

**Published:** 2008-07-31

**Authors:** Mark van Heijl, Jikke MT Omloo, Mark I van Berge Henegouwen, Olivier RC Busch, Hugo W Tilanus, Patrick MM Bossuyt, Otto S Hoekstra, Jaap Stoker, Maarten CCM Hulshof, Ate van der Gaast, Grard AP Nieuwenhuijzen, Han J Bonenkamp, John ThM Plukker, Ernst J Spillenaar Bilgen, Fibo JW ten Kate, Ronald Boellaard, Jan Pruim, Gerrit W Sloof, J Jan B van Lanschot

**Affiliations:** 1 Department of Surgery, Academic Medical Center, Amsterdam, The Netherlands; 2 Department of Surgery, Erasmus Medical Center, Rotterdam, The Netherlands; 3 Department of Clinical Epidiomiology, Biostatistics and Bioinformatics, Academic Medical Center, Amsterdam, The Netherlands; 4 Department of Nuclear Medicine, VU Medical Center, Amsterdam, The Netherlands; 5 Department of Radiology, Academic Medical Center, Amsterdam, The Netherlands; 6 Department of Radiotherapy, Academic Medical Center, Amsterdam, The Netherlands; 7 Department of Medical Oncology, Erasmus Medical Center, Rotterdam, The Netherlands; 8 Department of Surgery, Catharina Hospital Eindhoven, Eindhoven, The Netherlands; 9 Department of Surgery, Radboud University Medical Center, Nijmegen, The Netherlands; 10 Department of Surgery, University Medical Center Groningen, Groningen, The Netherlands; 11 Department of Surgery, Rijnstate Hospital, Arnhem, The Netherlands; 12 Department of Pathology, Academic Medical Center, Amsterdam, The Netherlands; 13 Department of Nuclear Medicine and PET research, VU Medical Center, Amsterdam, The Netherlands; 14 Department of Nuclear Medicine and Molecular Imaging, University Medical Center Groningen, University of Groningen, Groningen, The Netherlands; 15 Department of Nuclear Medicine, Academic Medical Center, Amsterdam, The Netherlands

## Abstract

**Background:**

Surgical resection is the preferred treatment of potentially curable esophageal cancer. To improve long term patient outcome, many institutes apply neoadjuvant chemoradiotherapy. In a large proportion of patients no response to chemoradiotherapy is achieved. These patients suffer from toxic and ineffective neoadjuvant treatment, while appropriate surgical therapy is delayed. For this reason a diagnostic test that allows for accurate prediction of tumor response early during chemoradiotherapy is of crucial importance. CT-scan and endoscopic ultrasound have limited accuracy in predicting histopathologic tumor response. Data suggest that metabolic changes in tumor tissue as measured by FDG-PET predict response better. This study aims to compare FDG-PET and CT-scan for the early prediction of non-response to preoperative chemoradiotherapy in patients with potentially curable esophageal cancer.

**Methods/design:**

Prognostic accuracy study, embedded in a randomized multicenter Dutch trial comparing neoadjuvant chemoradiotherapy for 5 weeks followed by surgery versus surgery alone for esophageal cancer. This prognostic accuracy study is performed only in the neoadjuvant arm of the randomized trial. In 6 centers, 150 consecutive patients will be included over a 3 year period. FDG-PET and CT-scan will be performed before and 2 weeks after the start of the chemoradiotherapy. All patients complete the 5 weeks regimen of neoadjuvant chemoradiotherapy, regardless the test results. Pathological examination of the surgical resection specimen will be used as reference standard. Responders are defined as patients with < 10% viable residual tumor cells (Mandard-score).

Difference in accuracy (area under ROC curve) and negative predictive value between FDG-PET and CT-scan are primary endpoints. Furthermore, an economic evaluation will be performed, comparing survival and costs associated with the use of FDG-PET (or CT-scan) to predict tumor response with survival and costs of neoadjuvant chemoradiotherapy without prediction of response (reference strategy).

**Discussion:**

The NEOPEC-trial could be the first sufficiently powered study that helps justify implementation of FDG-PET for response-monitoring in patients with esophageal cancer in clinical practice.

**Trial registration:**

ISRCTN45750457

## Background

Worldwide, esophageal cancer is sixth on the list of cancer related mortality causes. In the Western world the total incidence of esophageal cancer is rising, mainly as the result of a six fold increase in the incidence of adenocarcinoma over the last decades. [1] In the Netherlands approximately 1,500 new patients are annually diagnosed with esophageal cancer.

Surgical resection is currently the preferred curative treatment for esophageal cancer. In the Netherlands 300 – 400 patients per year are candidates for surgery with curative intent. [2] Even if esophageal cancer resection is only performed with curative intent, in approximately 30% of these patients the resection is microscopically irradical and 5-year survival rate among all operated patients is only 30–40%.[3,4]In order to enhance both locoregional and systemic tumor control, neoadjuvant treatment has been introduced. Internationally, many institutes apply neoadjuvant chemoradiotherapy to improve long term outcome, especially after the publication of a meta-analysis with favorable results comparing neoadjuvant chemoradiotherapy followed by surgery versus surgery alone. [5] However, in a proportion of patients insufficient objective response is achieved. These non-responding patients do not benefit from neoadjuvant therapy, but do suffer from toxic side effects like hair loss, nausea/vomiting, bone marrow depression (with risk of septic complications) and radiation-induced esophagitis (with further weight loss). This negative impact on the general condition of the patient probably leads to an increased perioperative morbidity and protracted postoperative recovery. Moreover, prolonged but ineffective preoperative treatment will inevitably delay appropriate surgical therapy. This down-side of neoadjuvant chemoradiotherapy in non-responders limits its general application in current esophageal cancer treatment. It underlines the need for early identification of non-responders.

Recently, the favorable effect of neoadjuvant chemoradiotherapy was confirmed in a Dutch phase-II trial, which tested a regimen of five weekly cycles of chemotherapy (paclitaxel and carboplatin) and 23 sessions of radiotherapy (daily dose of 1.8 Gy to a total dose of 41.4 Gy) in a group of 54 patients. The pathological complete response rate was 25%, and an additional 36.5% had less than 10% vital residual tumour cells. The remaining patients showed no response.[6] Because of these promising results, this same regimen will now be tested in a randomized, multicenter, Dutch phase-III trial, which offers an ideal opportunity to assess the accuracy of 18F-fluoro-deoxyglucose (FDG) positron emission tomography (PET) and spiral computed tomography-scan(CT) for the early prediction of non-response in the multimodality treatment arm.

At present, there are no clinical pretreatment characteristics (e.g. dysphagia), nor any biochemical or pathological markers that predict the response of an individual tumor to neoadjuvant chemoradiotherapy. Conventional imaging techniques for monitoring non-surgical therapy, such as CT and endoscopic ultrasound (EUS), are based on morphologic imaging. General restrictions of these methods include difficulty in distinguishing viable tumor from necrotic or fibrotic tissue and delay between cell kill and tumor shrinkage. [7] Nevertheless, CT-scanning is still widely applied for this purpose, partly because of its wide availability. EUS is hardly used for response assessment because of its limited accuracy and poor reproducibility. Moreover, EUS is discomfortable and not always feasible, especially in case of postradiation esophagitis or severe stricturing. [8]

A more accurate diagnostic modality that would be able to predict tumor response early in the course of neoadjuvant treatment is considered of crucial importance. FDG-PET has been shown sensitive in malignant lymphoma and non-small cell lung cancer for early assessment of tumor response to therapy and it has achieved promising results in several other malignancies (e.g. breast cancer, head and neck cancer). [9-12] In esophageal cancer the evaluation of FDG-PET for early monitoring of non-surgical therapy response is described in several small studies and in one large phase II trial, with promising results. [13-18] In this last trial non-responders discontinued chemotherapy after two weeks while responders underwent a total of 14 weeks of chemotherapy. Results show that PET is able to identify all patients who appear to be histopathological responders after resection. A large study investigating the ability of FDG-PET to identify responders to chemoradiation therapy has not yet been performed. Therefore, a large study with sufficient power to determine whether PET-scan may improve differentiation between responding and non-responding esophageal tumors early in the course of chemoradiation therapy is needed.

## Methods/design

### Study objectives

The objective of the NEOPEC-trial is to assess the accuracy and negative predictive value of FDG-PET and CT-scan for the early prediction of non-response to preoperative chemoradiotherapy in patients with potentially curable esophageal cancer. These parameters are defined as primary endpoints.

Secondly, the correlation between tumor response as predicted by FDG-PET and CT-scan or as measured by histopathologic assessment in the resection specimen and long term survival will be determined. This correlation will be used as a secondary endpoint.

### Study design

In September 2004 a multicenter randomized phase-III trial (175 patients in each arm) was initiated (CROSS trial) in the Netherlands, comparing neoadjuvant chemoradiotherapy followed by surgery with surgery alone in patients with potentially curable esophageal cancer. Long term survival and quality of life are the primary endpoints in this therapy-related trial. (ISRCTN80832026)

**Figure 1:**
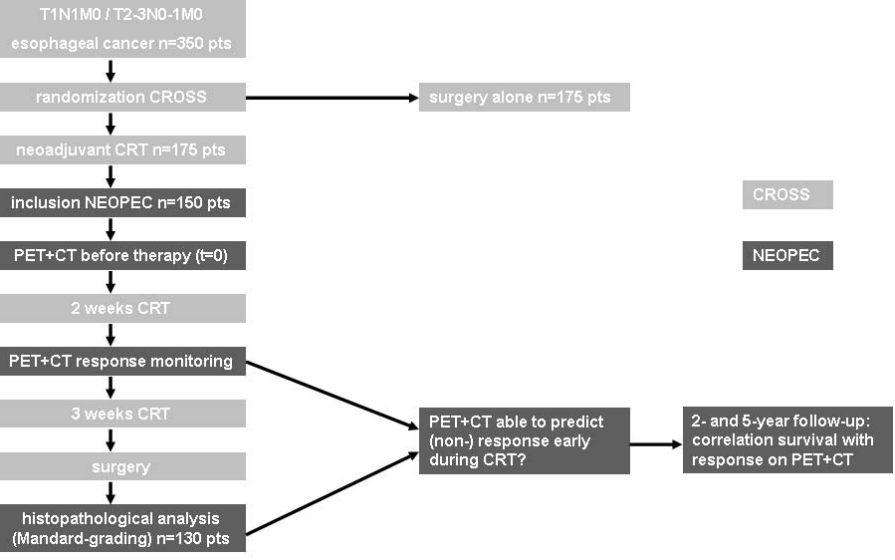
**integration NEOPEC-trial within CROSS-trial**. CRT chemoradiotherapy, PET positron emission tomography, CT computed tomography.

The present research proposal comprises serial FDG-PET and CT-scan before and during neoadjuvant therapy in the multimodality treatment arm of the randomized trial. Finally, resection specimens will be assessed for tumor response. Figure 1 describes the logistic integration of the NEOPEC-trial within the CROSS-trial.

### Patient selection

Patients with histologically proven squamous cell carcinoma or adenocarcinoma of the esophagus or gastro-esophageal junction will undergo extensive preoperative staging, including spiral CT-scan of the chest and abdomen. The tumor must not extend more than 2 cm into the gastric cardia. Longitudinal tumor length must not exceed 8 cm, radial size must not exceed 5 cm. cT1N0 tumors are not eligible. In the absence of local irresectability and/or distant dissemination patients with an acceptable general condition (ECOG performance status 0, 1, 2; weight loss < 10%) will be invited to participate in the randomized trial. After written, voluntary, informed consent and stratification for tumor type, treatment center, clinical lymph node status, and WHO performance the patients will be randomized between the two treatment-arms (neoadjuvant chemoradiation followed by surgery versus surgery alone). Patients who are randomized for the neoadjuvant arm will consequently be asked to participate in the NEOPEC-trial.

### Concurrent chemoradiotherapy CROSS-trial

Over a 5 week period concurrent chemoradiotherapy is applied on an outpatient basis.

Paclitaxel (50 mg/m2) and carboplatin (area-under-curve = 2) are administered by i.v. infusion on days 1, 8, 15, 22, and 29. Additional medication consists of dexamethason, clemastine, ranitidine, and ondansetron. Vital signs are monitored every fifteen minutes during paclitaxel administration.

External beam radiation with a total dose of 41.4 Gy is given in 23 fractions of 1.8 Gy, 5 fractions a week, starting the first day of the first cycle of chemotherapy. Radiation therapy will be delivered using a multiple field technique. All patients will undergo 3-D planning using a 3-D conformal technique.

### Surgery

Surgery, i.e. either transhiatal (1 field lymphadenectomy) or transthoracic (2 field lymphadenectomy) esophagectomy including lymph node dissection around the celiac axis, will be performed within 6 weeks of randomization (surgery alone arm) or within 6 weeks after completion of the chemoradiation (multimodality treatment arm). For esophageal surgery a clear correlation has been shown between hospital volume and quality of care. Therefore, only centers which perform at least 10 esophagectomies per year participate in this trial.

### Evaluation of tumor response by FDG-PET and spiral CT

PET-scan and CT scan will be performed at baseline and at day 15. FDG uptake in the tumor will be assessed semi quantitatively. PET-scans will be performed in several PET centers, using standardized acquisition and image reconstruction, with centralized data-analysis (VUMC PET data-analysis center) and after inter institute calibration of scanners. [19,20]

For quantitative analysis of FDG uptake the standardized uptake value (SUV) and the simplified kinetic method will be assessed using previously validated methodology. [9,10,12,20-22] Serum glucose levels will be measured using the standard hexokinase method. Fractional changes of FDG-uptake between baseline and early during treatment will be correlated with tumor response in the resection specimen. Further ROC analysis will be performed in order to obtain an optimal cut-off value of decrease in FDG uptake for the prediction of non-responders.

A target CT will be performed using a multi-slice CT scanner (minimal four-slice CT-scanner) after administration of 100 ml low osmolar intra venous contrast medium. After a 25 second delay a spiral scan CT is performed targeted at the esophagus and esophagogastric junction including the maximal extension of the tumor (maximum slice thickness 3 mm, maximum pitch ≤ 1.5, 150 mAs) with inferior-superior scanning. Scan settings concerning spatial resolution and contrast timing will be identical for pre- and post-treatment CT-scans. The tumor area will be calculated quantitatively by 3D volume measurement and multiplication of the greatest tumor diameter and the greatest perpendicular distance in the axial plane, as this is the clinically accepted method. [23,24] A decrease in tumor area or tumor volume of at least 50% is defined as tumor response. However, as for the FDG-PET data, ROC analysis will be performed to assess the optimal cut-off value for tumor response.

### Evaluation of tumor response in the resection specimen: reference standard

The 'reference standard' of the post-chemoradiation disease stage will be based on the histology in the surgical resection specimen. Treatment response will be classified according to the Mandard-score which is based on the percentage of viable residual tumor cells in relation to fibrosis/necrosis. [25] Responders are defined as patients with < 10% viable residual tumor cells; non-responders are defined as patients with > 10% viable residual tumor cells.

### Analysis

The accuracy of early FDG-PET and CT-scan in distinguishing responders from non-responders will be determined by using ROC-analysis to show the effect of different thresholds in early response to identify non-responders. Additional analyses will focus on the best cut-off points for PET- and CT-measurements to obtain the most effective approach for costs and quality of care.

### Sample size calculation

The sample size calculation is based on two considerations. First, to compare the accuracy between PET and CT in distinguishing responders from non-responders, the area under the ROC curve (AUC) will be used. Based on results of a previously publicized meta-analysis, the expected difference in AUC between PET and CT is 0.87 vs. 0.54. [7] To detect such a difference in an unpaired design, approximately 80 patients are required (with a power of 80% and a significance level of 5%), assuming that the proportion of responding patients is 50% (i.e. 40 responders in a group of 80 patients). [6,26]

Second, to be useful in clinical practice the best strategy to measure early treatment response (supposedly PET) should have a sufficiently high negative predictive value. At this moment, it is anticipated that the negative predictive value should be at least 0.80. Based on an expected sensitivity (true responders correctly identified by PET) of 95% and specificity (true non-responders correctly identified by PET) of 70%, 130 patients are needed to ensure that the lower limit of the exact 95% confidence interval around the negative predictive value will not reach below 0.80 (expected value of negative predictive value is then 0.93; 95% CI 0.83 to 0.99). The total number of 130 patients is based on the assumption that the proportion of responders will be 50%.

Therefore, a sample size of 130 evaluable patients is needed to answer both primary research questions. Anticipating a drop-out of 20 patients for various reasons after initial inclusion, we plan to include a total number of 150 patients in 3 years.

### Economic evaluation

The use of FDG-PET and CT-scan in all patients will increase costs however early discontinuation will reduce the costs of neoadjuvant treatment in those timely detected as non-responders. Therefore, the economic evaluation will be designed as a cost-effectiveness study.

The reference strategy is neoadjuvant treatment in all patients, without imaging to predict early response. The alternative strategies are FDG-PET or CT-scan in all patients and early discontinuation of neoadjuvant treatment in patients who test as negative (non-response). We will compare the incremental costs of these alternative strategies and the associated changes in survival and quality of life, relative to the reference strategy.

#### Cost analysis

We will estimate the direct medical costs and indirect (time) costs for all strategies by documenting resource utilization by counting volumes and using unit prices. The relevant cost items include: FDG-PET, CT-scan, neoadjuvant chemoradiotherapy (including the management of associated morbidity), surgery (distinguishing between patients with and without neoadjuvant treatment), and after hospital discharge: out-patient consultations, out-of-hospital consultations and hospital re-admissions.

Volume data will be collected through patient-specific case record forms and data retrieval from hospital information systems. Standard unit prices will be derived from the Dutch guideline on unit costing for economic evaluations in health care, from actual unit costing in the participating hospitals related to Dutch Diagnosis Related Groups (DBC) billing system, from previous research, and from charges in case of volume data of lesser significance.[27] The unit costs derived from multiple centers will be averaged.

#### Data-analysis

A decision analytic approach will be used to compare the overall survival, quality of life and costs of the three strategies: neoadjuvant chemoradiotherapy in all patients, without prediction of treatment response, FDG-PET to predict tumor response, with early discontinuation of chemoradiotherapy in patients with non-response and CT-scan to predict tumor response, with early discontinuation in patients with non-response.

#### Ethical approval

The present study was approved by the Medical Ethics Committee of the Academic Medical Center in Amsterdam, the Netherlands, with reference number MEC 04/210.

## Discussion

Nearly half of the patients receiving neoadjuvant therapy for esophageal cancer show no or little tumor-response. They suffer from toxic side-effects of chemotherapy and radiation-induced esophagitis, while appropriate surgical treatment is delayed. For this reason, many patients could benefit greatly from a test able to distinguish responders from non-responders, early in the course of neoadjuvant therapy.

Conventional staging modalities (CT and EUS) appear not able to monitor early response sufficiently, probably due to difficulty in distinguishing viable tumor from necrotic or fibrotic tissue and delay between cell kill and tumor shrinkage.[7,8] It seems that another test is needed for this purpose.

When FDG-PET would be implemented for response monitoring in future clinical practice, a negative test (i.e. no response on PET) implies that chemoradiotherapy will be discontinued. If the negative predictive value of PET is not sufficiently high, this would too often lead to erroneous discontinuation of chemoradiotherapy in responders, incorrectly classified by PET as being non-responders. The balance in positive and negative consequences associated with false-negative and false-positive test results will eventually determine which negative predictive value is sufficiently high. Ultimately, the results of the randomized therapeutic trial, especially the additional therapeutic effectiveness and side-effects of chemoradiotherapy, will be used to find the optimal balance.

Until now, early response monitoring in patients with esophageal cancer with FDG-PET has been studied only in relatively small trials and one larger trail in which patients discontinued chemotherapy when identified as non-responder. [13-18] Results of a sufficiently powered study with fully standardized PET-procedures may help to justify implementation of FDG-PET for response-monitoring of chemoradiation therapy in clinical practice.

## Abbreviations

NEOPEC: NEOadjuvant therapy monitoring with PET and CT in Esophageal Cancer; CT: computed tomography; FDG: 18F-fluoro-deoxyglucose; PET: positron emission tomography; EUS: endoscopic ultrasound; WHO: World Health Organization; ECOG: Eastern Cooperative Oncology Group; ROC: Receiver-operating characteristic; AUC: area under the curve.

## Competing interests

The authors declare that they have no competing interests.

## Authors' contributions

MvH drafted the manuscript. JJBvL co-authored the writing of the manuscript. All other authors participated in the design of the study during several meetings and are local investigators at participating centers. All authors read and approved the final manuscript.

## Pre-publication history

The pre-publication history for this paper can be accessed here:

http://www.biomedcentral.com/1756-6649/8/3/prepub
